# 107 Genital Burn Injuries: A Nationwide Analysis of Patient Outcomes

**DOI:** 10.1093/jbcr/irae036.106

**Published:** 2024-04-17

**Authors:** Daniel Najafali, Michael Pozin, Suma Gangidi, Mukul Govande, Justin M Camacho, Quincy K Tran

**Affiliations:** Carle Illinois College of Medicine, Urbana, IL; Drexel University college of medicine, New york, NY; University of Maryland School of Medicine, Baltimore, MD; Carle Illinois College of Medicine, Urbana, IL; Drexel University college of medicine, New york, NY; University of Maryland School of Medicine, Baltimore, MD; Carle Illinois College of Medicine, Urbana, IL; Drexel University college of medicine, New york, NY; University of Maryland School of Medicine, Baltimore, MD; Carle Illinois College of Medicine, Urbana, IL; Drexel University college of medicine, New york, NY; University of Maryland School of Medicine, Baltimore, MD; Carle Illinois College of Medicine, Urbana, IL; Drexel University college of medicine, New york, NY; University of Maryland School of Medicine, Baltimore, MD; Carle Illinois College of Medicine, Urbana, IL; Drexel University college of medicine, New york, NY; University of Maryland School of Medicine, Baltimore, MD

## Abstract

**Introduction:**

Burn injuries to the genitals can lead to devastating injury with lifelong sequela that can impair function, aesthetics, and hygiene, directly impacting a patient’s quality of life. There is limited research investigating burn injuries to the genitalia. This study aims to characterize the outcomes of patients with burn injuries to their genitals.

**Methods:**

Burn encounters to the genitals were isolated from ABA Burn Care Quality Platform between January 2008 to December 2018. Cohorts were separated based on age: adult and pediatric. Descriptive statistics summarized patients’ demographics, injury characteristics, treatment course, and ultimate dispositions. Multivariable logistics regressions evaluated any complications, length of stay, and mortality.

**Results:**

A total of 7,096 patients had burn injuries to the genital region, of which 4,478 (63%) were adults. The majority of patients were male (66%), Caucasian (49%), and had a mean (SD) age of 31 (26) years. The most common cause of injury for adults was flame-based (34%), whereas scald injuries were responsible for 80% of the genital injuries for the pediatric population (p < 0.001). Median (IQR) TBSA burned was 18% [5-54%] for adults and 6% [3-15%] for pediatric cases (p < 0.001). When isolating the percent of body surface area that was directly impacting the genital region the median was 6% [2-17%]. Most patients had no complications (75%) and survived their burn injury (84%). Factors significantly associated with complications during treatment were pediatric patients (OR 2.0, 95%CI 1.68-2.30) and having a higher proportion of the burn concentrated to the genital region (OR 1.02, 95%CI 1.01-1.02). Longer lengths of stay were observed in black patients (OR 1.22, 95%CI 1.04-1.44) and patients with rash or skin infections (OR 18.9, 95%CI 12.4-29.8), whereas shorter lengths of stay were associated when burns were concentrated on the genitals (OR 0.97, 95%CI 0.97-0.98). Mortality was significantly associated with race, etiology of injury, TBSA, and a greater proportion of burn concentrated on the genitals.

**Conclusions:**

This study highlighted outcomes for burn injuries to the genital region and found that most patients are discharged. We observed that a greater burn concentration to the genital area significantly correlated with increased complications. Paradoxically, these cases were associated with shorter hospital stays. To better support patients with genital burns, we recommend the development of specialized post-discharge care protocols to mitigate potential complication and ensure optimal long-term outcomes.

**Applicability of Research to Practice:**

There is a gap in the literature regarding burn injuries sustained to the genitals and their management. Focusing on identifying populations at increased risk of poor outcomes will inform managing providers.

